# *In vivo* anthelmintic activity of *Eleusine indica* extracts against gastrointestinal nematodes of goats

**DOI:** 10.5455/javar.2023.j686

**Published:** 2023-09-24

**Authors:** Elsa Amarille Gonzaga1, Tiffany Velasco Taño, Loveille Jun Amarille Gonzaga

**Affiliations:** 1 Department of Veterinary Paraclinical Sciences, College of Veterinary Medicine, University of Southern Mindanao, Kabacan, Cotabato, Philippines; 2 College of Veterinary Medicine, University of Southern Mindanao, Kabacan, Cotabato, Philippines; 3 Department of Chemistry, College of Science and Mathematics, University of Southern Mindanao, Kabacan, Cotabato, Philippines

**Keywords:** Anthelmintic, *Eleusine indica*, gastrointestinal nematodes, goats, levamisole

## Abstract

**Objective:**

The study aimed to determine the potential anthelmintic activity of the ethyl acetate extract of *Eleusine indica* that will result in an effective reduction in fecal egg per gram (EPG) counts in naturally infected goats compared to the commercial anthelmintic levamisole.

**Materials and Methods:**

The experimental animals were 21 goats naturally infected with gastrointestinal nematodes. The goats were divided into groups that were given a single dose of *E. indica* extract. Five concentrations of *E. indica* were tested for anthelmintic activity: 100, 200, 300, 400, and 500 mg extract/kg body weight. Fecal sample collection was done before treatment, during the first treatment, and every week thereafter for 28 days post-treatment (dpt). A modified McMaster technique was used to determine the EPG of feces, and the mean efficacies of the extracts were compared with those of the commercial anthelmintic levamisole.

**Results:**

As early as 7 dpt, there was an observed reduction in the epg counts after the administration of *E. indica* extracts across all concentrations. Administering 500 mg of extract/kg body weight resulted in a maximum efficacy of 56.21%. However, the efficacy achieved was lower than that of levamisole (96.83%).

**Conclusion:**

The results show that the *E. indica* extract can reduce the fecal EPG counts of naturally infected goats, thus creating a potential natural anthelmintic that can be developed further.

## Introduction

Conditions that cause a reduction in feed intake and alteration of gastrointestinal motility and digestive flow cause inappetence and diarrhea in goats, resulting in a profit loss for goat raisers [1]. The most common constraint in small-ruminant farming is the infection of gastrointestinal nematodes, particularly in tropical and subtropical countries [2]. These parasites are accountable for weight loss, diarrhea, and anorexia.

The use of anthelmintic products is the usual mode of control for gastrointestinal parasites [3]. Commercial anthelmintics used include levamisole, albendazole, and ivermectin [4]. However, due to the repeated use of these anthelmintics, parasites have developed an increasing degree of resistance to these formulations [5]. Therefore, various alternative veterinary interventions are being made to help eradicate the problem, particularly through the use of natural anthelmintics.

Promising sources of these natural anthelmintics are medicinal plants and herbs [6]. Anthelmintic activity is generally associated with tannins, particularly condensed tannins and polymers of flavonoid units, as these increase postruminal protein availability and thereby reduce the consequences of gastrointestinal nematode parasitism [7].

*Eleusine indica *is an annual, erect, and monocot weed that belongs to the family Poaceae [8]. It contains flavonoids, schaftoside, and vitexin, both of which exhibit various bioactivities in studies, such as antibacterial, antidiuretic, anti-inflammatory, antiviral, and antifungal properties. In addition to flavonoids, tannins, and alkaloids are also present in *E. indica* extracts [9]. The phytochemical profile and bioactivities of identified compounds of *E. indica* show the potential that it could be used as an alternative to commercial anthelmintic products. It has been previously reported to exhibit *in vitro* anthelmintic activity against *Strongyloides stercoralis *[9]. Thus, this study aimed to determine the efficacy of *E. indica* leaf extract against gastrointestinal nematodes in naturally infected goats for its potential veterinary applications.

## Materials and Methods

The procedures used in the study were approved by the University of Southern Mindanao Institutional Animal Care and Use Committee DVM-23. Animal handling and research methods are all in compliance with Republic Act 8485, the Animal Welfare Act.

### Plant material and extract preparation

*Eleusine indica* leaves that were healthy and had no signs of damage, such as insect bites or discoloration, were collected in Kabacan, Cotabato, Philippines. The authentication of the samples was done at the University of Southern Mindanao Department of Biological Sciences. Extraction was based on a previously described process [10], with modifications to the extracting solvent. The plant material was washed with distilled water, dried at room temperature, and powdered using a grinder. The ground *E. indica* leaves (500 gm) were soaked in ethanol (3-l) for 48 h and filtered. The filtrate was dried in vacuo to obtain a crude extract. The crude extract was then separated into organic and aqueous layers using ethyl acetate and water, respectively. The organic layer (ethyl acetate) was used as the final extract in the study.

### Animals

Twenty-one goats were used as experimental animals in the study. The goats, between 3 and 5 months of age, were chosen regardless of sex or breed and were naturally infected with gastrointestinal nematodes. The goat management system at the Goat Project facility in Carmen, Cotabato, was adapted, where the animals belonged to the same herd in a semi-confined rearing system. The facility had an elevated indoor area with a wood-slatted floor where the animals were given time to roam around. Water, grass hay, and mineral salt were provided ad libitum. The diets provided met nutritional requirements and were free of anthelmintics.

### In vivo treatment

The modified McMaster method [11] was used during the preliminary fecal examination to determine the eggs per gram (EPG) of feces and identify naturally infected goats. Larval culture was done to identify the nematode species present. Goats with more than 500 EPG counts were used as experimental animals.

Twenty-one naturally infected goats were identified and were divided into seven homogeneous groups of three goats each. Oral administration was done for the treatments, extracts, and control. Goats in groups I–V were treated with a single dose of 100, 200, 300, 400, and 500 mg of extract per kg of body weight. Group VI was the positive control and was treated with a single dose of levamisole, 5 gm per 12 kg of body weight, while group VII was the negative control and was not subjected to any treatment. Fasting was done for about 12 h before the *E. indica* extract was given orally early in the morning. This was done to slow down gastrointestinal transit and improve the absorption of the orally administered drug [12].

### Parasitological examination

The collection of the fecal samples (3–5 gm) was done 7, 14, 21, and 28 days post-treatment (dpt). A correction factor based on a scale of 1–3 was used to score fecal samples with 1, normal pellets; 1.5, soft formed but the pellets did not separate; 2, soft but there is no formation of pellets; and 3, diarrheic [13]. A modified McMaster technique [11] was used for the fecal examination. The calculation of the % efficacy was done by dividing the difference between the post-treatment EPG and the pretreatment EPG by the pretreatment EPG and multiplying by 100.

### Statistical analysis

Results are expressed as mean ± SE of means. Statistical significance among treatments (*p *≤ 0.05) was compared using one-way analysis of variance and the Tukey honestly significant difference test.

## Results and Discussion

The mean EPG counts of goats in the different treatment groups are shown in Table 1. Among the naturally infected goats, *Haemonchus contortus* was the dominant gastrointestinal nematode. There was an observed reduction of EPG after administering *E. indica* in naturally infected goats 7 dpt. However, there was no further reduction in the EPG 14, 21, or 28 dpt.

Table 2 shows the efficacy of the extracts in each treatment. Within 7 dpt, group V had the highest efficacy among the extracts. However, this was lower than the commercial anthelmintic levamisole. The extracts reached a maximum efficacy of 56.21% with group V at 14 dpt, which was still lower than the efficacy of levamisole. The efficacy of the extracts then decreased until 28 dpt.

Statistical analyses show that from 7 to 28 dpt, *E. indica* extracts exhibited anthelmintic activity, as evidenced by their efficacy in comparison to the negative control. However, this anthelmintic activity was weak in comparison to that of levamisole. The phytochemicals present in the *E. indica* extract may explain this anthelmintic activity.

**Table 1.** The mean fecal EPG of naturally-infected goats treated with *E. indica *extract.

Group	dpt				
	0	7	14	21	28
I (100 mg/kg)	975 ± 75^a^	625 ± 88^a^	654 ± 76^a^	646 ± 42^a^	684 ± 65^a^
II (200 mg/kg)	1,367 ± 292^ab^	854 ± 157^a^	821 ± 138^a^	825 ± 188^a^	833 ± 147^a^
III (300 mg/kg)	1,733 ± 94^abc^	975 ± 14^ab^	975 ± 38^ab^	1,000 ± 38^a^	1,067 ± 44^a^
IV (400 mg/kg)	1,900 ± 101^abc^	1,025 ± 63^a^	1,000 ± 14^ab^	975 ± 29^a^	1,042 ± 51^a^
V (500 mg/kg)	1,225 ± 101^bc^	571 ± 34^a^	529 ± 18^ab^	579 ± 33^a^	654 ± 70^a^
VI (levamisole)	1,142 ± 68^c^	38 ± 22^b^	17 ± 17^c^	13 ± 13^b^	46 ± 29^b^
VII (untreated)	742 ± 118^c^	733 ± 121^a^	725 ± 115^b^	717 ± 106^a^	725 ± 88^a^

*Mean values in the same column followed by the same superscript are not significantly different (*p* < 0.05).

**Table 2.** The mean efficacy of *E. indica* extract in different groups.

Group	dpt			
	7	14	21	28
I (100 mg/kg)	36.22 ± 5.66^c^	33.14 ± 4.12^c^	33.60 ± 1.15^d^	30.01 ± 2.57^c^
II (200 mg/kg)	36.76 ± 2.55^c^	38.83 ± 3.67^bc^	39.91 ± 1.64^cd^	38.16 ± 2.37^bc^
III (300 mg/kg)	43.51 ± 2.22^bc^	43.60 ± 2.01^bc^	42.15 ± 2.07^cd^	38.37 ± 0.94^bc^
IV (400 mg/kg)	46.02 ± 2.02^bc^	47.11 ± 2.46^bc^	48.30 ± 3.89^bc^	45.15 ± 0.32^b^
V (500 mg/kg)	52.90 ± 3.83^b^	56.21 ± 3.83^b^	52.43 ± 3.89^b^	46.63 ± 2.97^b^
VI (levamisole)	96.83 ± 1.71^a^	98.48 ± 1.52^a^	98.79 ± 1.21^a^	95.98 ± 2.68^a^
VII (untreated)	1.18 ± 3.47^d^	1.92 ± 5.75^d^	3.10 ± 1.57^c^	1.18 ± 3.47^d^

*Mean values in the same column followed by the same superscript are not significantly different (*p* < 0.05).

The ability of *E. indica* to reduce EPG can likely be attributed to the bioactive components present. Extracts of *E. indica* using different extracting solvents have been able to exhibit varying degrees of antibacterial, antioxidant, and cytotoxic properties. These bioactivities have been attributed to the phenols present in *E. indica* [14]. The presence of flavonoids and alkaloids was found in the ethanol extract of *E. indica*, but tannins were only present in the methanol extract. Both ethanol and methanol extracts exhibited antibacterial activity. Moreover, the extract also exhibited *in vitro* anthelmintic activity against *S. stercoralis* [9].

The presence of condensed tannins in plant extracts has been associated with being the main secondary metabolite responsible for anthelmintic activity [6]. It is suggested that condensed tannins have a marked effect on the subsequent development of nematode larvae. Condensed tannins are not absorbed in the digestive tract and thus become concentrated in the feces [15]. This affects the hatchability of the nematode eggs, leading to lower pasture contamination. This means that the direct anthelmintic effects of tannins are limited to the developing stages and not adult nematodes.

Aside from tannins, flavonoids and phenols have also been attributed to exhibiting anthelmintic activity. Flavonoids and cinnamic acid derivatives such as quercetin, caffeic acid, and coumaric acid have been reported to inhibit the hatching of *H. contortus* at concentrations as low as 1 mg/ml. Phenolic compounds, particularly hydroxycinnamic acid and flavonoid derivatives, are associated with anthelmintic activity [16]. It was suggested that phenolic compounds permeate the cuticular layers of either eggs or infective larvae of the parasite [17].

Alkaloids may also be attributed to anthelmintic activity. Berberine, harmaline, and piperine are alkaloids reported to inhibit the egg-hatching of gastrointestinal nematodes in goats. Moreover, berberine exhibited larvicidal activity, significantly inhibiting the motility of infective larvae [18]. These results show that berberine was effective in different stages of the nematode. This corroborates the previously reported anthelmintic activity of berberine against third-stage larvae species of the genus *Strongyloides* [19]. Steroidal alkaloids and aminoglycosides alter nitrate generation and the sugar that reaches the stomach, resulting in unfavorable conditions for parasite development [20]. Moreover, the piperidine alkaloids pelletierine and arecoline may cause parasite paralysis due to the inhibition of acetylcholine receptors [21].

Secondary metabolites from plant extracts have various mechanisms and modes of action responsible for anthelmintic activity. Moreover, the efficacy depends on the different developmental stages of the parasite. These can inhibit hatchability and larval motility, damage the cuticle, interfere with feeding, and harm the growth and reproduction of helminths [18]. It should be noted that despite the promise of anthelmintic activity, some plant extracts may have no effect *in vivo* [22]. The failure of *in vivo* tests may be due to the interaction of the secondary metabolites with the diet and the host metabolism, leading to a lower and, thus, insufficient concentration of active compounds that could elicit anthelmintic activity.

The use of ethanol for the crude extract and subsequently ethyl acetate for the final extract implies a low concentration of tannins in the extract, if there were any. As a result, the anthelmintic activity exhibited by *E. indica* can be attributed to the flavonoids and phenols dominantly present in the extract. The flavonoids present are hypothesized to inhibit the hatching of *H. contortus* and the phenols disabled the eggs by permeating the cuticular layers. In contrast, the alkaloids may have inhibited acetylcholine receptors, leading to helminth paralysis.

There were no apparent negative side effects on the overall health and well-being of the goats due to the extract. However, it was reported that although organ weights were unaffected, there was slight inflammation of the liver, spleen, lungs, kidneys, and brain of adult albino Wistar rats administered low doses of *E. indica* extract. Upon administering higher doses, moderate inflammation was recorded in the spleen and lungs, and moderate interstitial fibrosis was noted in the lungs. The extract was administered on alternate days for 28 days [23]. This might have caused such results, given the prolonged administration of the extract and the potential overdose from the extract.

Nevertheless, the anthelmintic activity against gastrointestinal nematodes in naturally infected goats shows that the administration of *E. indica* extracts contains sufficient amounts of the bioactive compounds and, as a result, is effective *in vivo*.

## Conclusion

The study results show that the leaf extract of *E. indica* has potential *in vivo* anthelmintic activity. However, this activity is lower than that of the commercial anthelmintic levamisole. Nevertheless, the anthelmintic activity of *E. indica* is enough to warrant that it has the potential to be developed as a naturally-sourced anthelmintic.

## List of Abbreviations

dpt: days post-treatment; *E. indica*: *Eleusine indica*; EPG: eggs per gram of feces; g: gram; kg: kilogram; mg: milligram.
